# The IgE response to *Ascaris* molecular components is associated with clinical indicators of asthma severity

**DOI:** 10.1186/s40413-015-0058-z

**Published:** 2015-03-04

**Authors:** Emiro Buendía, Josefina Zakzuk, Dilia Mercado, Alvaro Alvarez, Luis Caraballo

**Affiliations:** Institute for Immunological Research, University of Cartagena, Cartagena, Colombia; Foundation for the Development of Medical and Biological Sciences (Fundemeb), Cartagena, Colombia; Department of Microbiology, Faculty of Medicine, University of Cartagena, Cartagena, Colombia

**Keywords:** Asthma severity, *Ascaris*, House dust mites, IgE, The tropics, Lung function, Atopy, *Blomia tropicalis*, *Dermatophagoides pteronyssinus*, Ascariasis, Poverty, Urban

## Abstract

**Background:**

Asthma is a common chronic disease worldwide and *Ascaris lumbricoides* infection (ascariasis) is frequent in tropical regions. However, the effect of ascariasis on asthma severity has not been sufficiently explored. We sought to evaluate the influence of the IgE immune response to *Ascaris* extract and purified house dust mites (HDM) and *Ascaris* allergens on indicators of asthma severity in patients living in the tropics.

**Methods:**

Asthmatic patients from Cartagena, Colombia were recruited. Clinical assessment included questionnaires, physical examination, allergy skin tests, spirometry, parasite stool examination and IgE antibody measurements. Asthma was diagnosed by a physician according to validated criteria. Indicators of severity were occurrence of severe dyspnea episodes, night awakenings events, > 4 emergency room (ER) visits and hospitalizations during the last year. Specific IgE to Der p 2, *Ascaris spp.*, *Blomia tropicalis* and *Dermatophagoides pteronyssinus* extracts was determined by ImmunoCap. IgE to tropomyosins (Asc l 3, Blo t 10 and Der p 10), Blo t 5 and Asc s 1 was detected by ELISA. Logistic regression analyses were used to explore the relationships between sensitization and indicators of asthma severity.

**Results:**

After adjustment for HDM sensitization, *Ascaris* sensitization remained associated with severe dyspnea (aOR: 1.90, 95% CI: 1.08 - 3.34, p = 0.03) and > 4 ER visits (aOR: 2.23, 95% CI: 1.15 - 4.30, p = 0.02). We also found that sensitization to the species specific markers Blo t 5 and Asc s 1, as well as the cross-reactive tropomyosins of *D. pteronyssinus* and *Ascaris* were associated with > 4 ER visits. Der p 2 sensitization was associated with bronchodilator responsiveness (aOR: 2.24: 1.25-4.02, p = 0.01). Remarkably, significantly higher IgE levels to HDM species specific allergens were found in *Ascaris* sensitized patients.

**Conclusions:**

In this tropical population, IgE sensitization to *Ascaris* and the cross-reactive tropomyosins was frequent and associated with clinical indicators of asthma severity. The significant relationship between sensitization to the nematode-specific marker Asc s 1 and ER attendance supports these findings. Moreover, ascariasis increases the human IgE responses to HDM specific allergens.

## Background

Asthma is a common chronic respiratory disease globally distributed, including urban areas of low to middle income countries [1]. The majority of asthma cases are associated with atopy, but clinical presentation may vary under the influence of other risk factors. For example, the severity of symptoms could be influenced by optimal medication prescription, adherence to therapy, environmental exposures and genetic factors.

In the Tropics asthma cases are strongly associated with sensitization to house dust mites (HDM) such as *Blomia tropicalis* and *Dermatophagoides pteronyssinus,* which are the most important risk factors for this disease, probably as a result of the permanent exposure to these allergen sources. In addition, ascariasis, caused by the nematode *Ascaris lumbricoides,* is also frequent and several outcomes resulting from the coexistence of these problems have been reported, suggesting an important influence of this infection on the pathogenesis, prevalence, diagnosis and treatment of asthma [2,3].

Ascariasis is the most frequent soil-transmitted helminthiasis [4,5]. In South American countries it is more common in rural [6-9] than urban settings [10,11]. Its influence on asthma is still controversial; population surveys in lightly-infected urban communities have shown that the infection is a predisposing factor for allergen sensitization [12,13] and asthma symptoms [14-16], while studies in heavily-infected rural populations have found that ascariasis may protect from some allergic symptoms [17,18]. Therefore, more research is needed to understand the relationship between helminth infections and allergy, particularly to explore the potential HDM-independent effect of ascariasis on the presence and severity of allergy symptoms.

Active ascariasis may exacerbate asthma symptoms [10], in some patients as a consequence of larvae migration to the lung [19]. Also, a positive association between infection/sensitization and severity of asthma symptoms has been described [12,20]. Ascaris infection promotes Th2 responses, increasing total and specific IgE [21,22] and there is evidence that the strong IgE response is associated with asthma [13,23]. Since HDM share allergenic components with Ascaris [24] a clinical role by increasing the antibody mediated allergic response is possible [25]. In addition there is evidence from animal models that ascariasis may increase the IgE responses to bystander antigens, suggesting another mechanism underlying its influence on allergic symptoms. However, the relationship between this IgE hyperresponsivenes and the severity of asthma has not been sufficiently investigated, an important gap considering that about half of people live in the tropics.

Because of the permanent co exposure to HDM allergens and *Ascaris* infection and the cross reactivity between allergens from these sources, the evaluation of the potential role of IgE response to *Ascaris* on asthma should include, in addition to adjusting for confounders, the use of both, species-specific and cross reacting allergens. In this study, we aimed to investigate the role of *Ascaris* and purified allergenic components sensitization on indicators of asthma severity in patients living in a tropical Caribbean city. We also evaluated the influence of sensitization to a nematode specific marker on the strength of the IgE response to mite-specific allergens.

## Methods

### Design, location and study population

This is a cross-sectional study to analyze the relationship between *Ascaris* sensitization and asthma severity in 313 patients. The study population is from Cartagena de Indias, a tropical city in the Caribbean Coast of Colombia (10° 23' 59″ North, 75° 30' 52″ West) with an average annual temperature of 28°C and 80% of relative humidity. Most inhabitants are poor according to governmental indexes that assess type of housing, overcrowding (three or more people per bedroom), access to basic services, income and school attendance. This socioeconomic stratification ranges from 1 to 6 and 90% of the study population is grouped in the lowest strata (1 to 3) [26] and shared environmental conditions. The genetic background resulted from racial admixture between Native Americans, Spaniards, and an important proportion (37.9%) of African ancestry [27]. The study was approved by the Ethics Committee of the University of Cartagena (Cartagena, Colombia). Signed informed consent was obtained from patients or their parents.

### Eligibility criteria and enrollment procedures

Subjects attending to five public primary health care centers and the University Hospital were screened for eligibility by physicians of the research staff between June 2010 and March 2011. These centers serve the lowest social strata in the city. Eligibility criteria were: subjects in the age range of 8 to 70 years who answered affirmatively to the question: *Have you ever been diagnosed with asthma?* Inclusion in the study depended on the confirmation of asthma diagnosis made by the physician. Patients with chronic obstructive pulmonary disease or another chronic respiratory co-morbidity were excluded as well as those patients belonging to the highest socio-economical strata of the city (4 to 6). Patients received an explanation about the investigation and signed a written informed consent to participate.

### Asthma diagnosis and indicators of severity

Eligible subjects were further interviewed and asthma diagnosis was confirmed in those with at least two respiratory symptoms (cough, wheezing, dyspnea, and nocturnal cough/wheezing/dyspnea) or a history of recurrent asthma attacks. These questions were done by staff physicians following a validated questionnaire [28,29]. In addition, using the same questionnaires presence of symptoms such as severe dyspnea, night awakenings and number of ER visits and hospitalizations in the last 12 months were assessed.

### Skin prick test

Skin prick test (SPT) was done in the forearm with a battery of allergen extracts (kindly supplied by Leti; Madrid, Spain) including: *B. tropicalis*, *D. pteronyssinus, Periplaneta americana*, *Penicillium spp*., *Aspergillus fumigatus*, *Alternaria alternata, Artemisia artemisoflia,* dog and cat epithelium extracts, histamine phosphate 10 mg/mL as positive control and allergen diluent as negative control. The test was considered positive if the mean diameter of the wheal at 15 minutes was > 3 mm than the negative control.

### Assessment of lung function

Spirometry was performed with a Microlab spirometer (Carefusion Corporation, USA) following the American Thoracic Society recommendations [30]. Height and weight were measured; patients were instructed to avoid use of short-acting bronchodilators for at least 12 hours before testing. The best forced expiratory volume in one second (FEV1) and forced vital capacity (FVC) were selected for data analysis of FEV1 and FEV1/FVC ratio. To evaluate bronchodilator responsiveness (BDR), spirometry was performed before and 15 minutes after receiving 200 μg (2 puffs) of inhaled salbutamol.

### Stool samples and parasitological examination

Parasitological analyses were done using 0.85% saline solution and lugol staining; counting helminth eggs were done by the Kato Katz method using a commercial kit (Copro Kit, C&M Medical, Campinas, Brazil). The results were expressed as egg per gram of feces (e.p.g.). The presence of eggs from geohelminths or parasite visualization was considered diagnostics of active infection. Antecedents of helminthic infection were asked by a questionnaire.

### Cloning and expression of recombinant allergens

Asc s 1 (ABA-1) is an allergen of *Ascaris* spp. that has been found only in nematodes; it is a member of the polyprotein allergen/antigens with fatty acid-binding properties [31,32]. It has been used as serological marker of *Ascaris* infection [33-35] and due to its lack of cross-reactivity with mite allergens [36] is very useful as a nematode specific diagnostic reagent. Asc l 3 is the tropomyosin from *Ascaris lumbricoides*, cross-reactive with mite orthologs [37]. Recombinant Asc s 1 (kindly supplied by Malcolm Kennedy, University of Glasgow) and Asc l 3 were obtained as previously described [24,38]. Blo t 5 is a major allergen from *B. tropicalis*; its lack of cross-reactivity with *Ascaris spp*. has been demonstrated experimentally [39]. Its nucleotide sequence was amplified from a cDNA library of *B. tropicalis* and the fragment was cloned into a pET100 vector, expressed in BL21 (DE3) cells and purified as a 6xhis tagged protein; its sequence had 100% of identity with the one reported by Arruda *et al.* [O96870] [40].

Codon-optimized gene sequences of the tropomyosins Blo t 10 [ABU97466.1] and Der p 10 [AAB69424.1] were synthetized and subcloned into pET45b + vector by Genscript (Piscataway, USA). For protein expression, competent *Escherichia coli* BL21 (DE3) cells were transformed by electroporation and selected on Luria broth (LB) agar plates containing ampicillin. A saturated *E. coli* culture was inoculated into LB medium with ampicillin, grown at 37°C until it reached an OD_600_ of 0.5 and induced with 1 mM Isopropyl-1-β-thiogalactopyranoside. After 5 h of shaking at 37°C, cells were centrifuged at 3500 rpm during 30 min at 4°C. Induced cultures were re-suspended in native buffer (50 mM NaH_2_PO_4_ 300 mM NaCl) incubated with lysozyme (1 mg/ml) and then sonicated. Lysates were incubated with Ni-NTA resin (Invitrogen) for one hour, washed with native buffer plus 20 mM imidazole and eluted with native buffer plus 250 mM imidazole as a 6xHis-tagged protein.

### Quantification of total and specific IgE

Blood samples were taken by venipuncture at the first visit to medical office using anticoagulant-free tubes to obtain serum for antibody determinations. Serum total IgE and specific IgE levels against *B. tropicalis, D. pteronyssinus,* Der p 2 and *A. lumbricoides* were determined by ImmunoCap system (Phadia100, Thermo, Sweden). A cut-off value of 0.35 kU/L was used to consider a test as positive. Specific IgE levels to Asc s 1, Blo t 5, Asc l 3, Blo t 10 and Der p 10 were measured by indirect ELISA, as described previously [24,36]. The cutoff for Blo t 5 and Asc s 1 was defined as an optical density (OD) value greater than 0.130. In the case of the three tropomyosins, the OD cut-off value was 0.156.

### Clinical phenotypes and other outcomes

The following outcomes were evaluated: **Indicators of asthma severity**: occurrence of severe dyspnea episodes, night awakenings events, >4 ER visits and hospitalizations during the last year. **BDR**: a change greater than 12% in the predicted FEV1 value after bronchodilator administration. **HDM sensitization**: A positive IgE result to *B. tropicalis* or *D. pteronyssinus* extracts. **Ascaris sensitization**: A positive IgE result to *Ascaris* extract. **Atopy**: at least one positive SPT to any of the tested allergens.

### Statistical analysis

Most analyses were done using SPSS version 13.0 (Chicago, IL, USA). Frequency rates and their 95% confidence intervals (CI) were obtained with Epidat 3.1 (Xunta de Galicia, PAO/WHO). Total and specific IgE values were not normally distributed and they were therefore reported as the median value and its inter-quartile range, except total IgE (geometric mean). Mann–Whitney *U* (MW) test was used for comparison of continuous variables. Differences between proportions were analyzed by Pearson chi-squared test (or Fisher exact test when appropriate). Chochran-Armitage test was used for trend association analysis.

Univariate and multivariate binary logistic regression were used to analyze the relationships of exposures and outcomes. Sensitizations to HDM, *Ascaris* or the recombinant allergens were included in the models as predictor/exposures. Indicators of disease severity and lung function parameters were analyzed as outcomes. Significant associations in the univariate analysis were further explored in multivariate models including age, gender, socio-economical strata, tobacco consumption and co-habitation with a smoker as covariates. Additionally, HDM sensitization or atopy was included as covariates in separate models. Crude (OR) and adjusted odds ratios (aOR), their 95% CI and p-values were calculated.

### Sample size and power

Power was calculated assuming an independent case–control design and expressing the alternative hypothesis as Odds Ratio (ψ). For a given risk factor the calculation was done by setting type I error probability at α = 0.05, number of cases (n), probability of exposure among controls (ρ0), and the ratio of controls/cases (m) [41].

A priori calculations of study power were done with published data of 57.8% of Ascaris sensitization in our population [42] and prevalence rates of asthma severity indicators, estimated within a national epidemiological survey [28]. With a sample size n = 330, assuming odds ratio of 2, power values > 0.8 were obtained to detect associations between Ascaris sensitization and any of these outcomes.

## Results

### Characteristics of the study population

**Figure 1:**
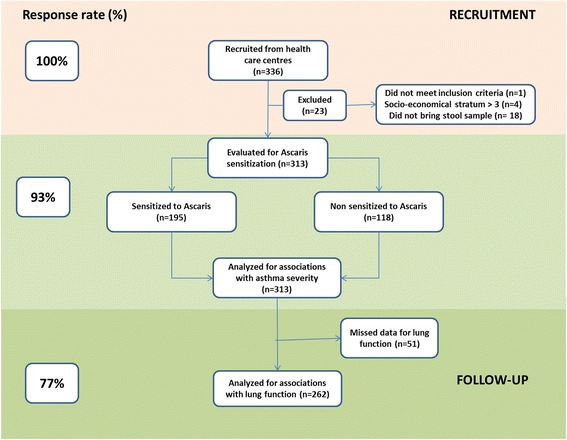
**Flow-chart of data collection and response rates in the study.** Responses rates (left boxes) are shown as a percentage, relative to the total number of recruited individuals. Exclusion criteria, applied during recruitment, and missing data cases (right boxes) are also detailed.

From 336 recruited individuals, one did not meet criteria for asthma diagnosis and 22 were excluded (Figure 1). Socio-demographic characteristics and severity of symptoms of the excluded subjects were similar to those that continued in the study. In total, 313 patients were included, *Ascaris* sensitization was evaluated in all of them but lung function data were obtained from 262. Reasons for not performing the test were use of β2 agonist drugs in the previous 12 hours and no collaboration of the patient to follow the instructions.

Power estimates for the analyzed outcomes were as follows: nocturnal awakenings: 0.60 (59.6% of exposed subjects in cases vs. 58.7% in control group), severe dyspnea: 0.75 (69.2% of exposed subjects in cases vs. 54.5% in control group), hospitalizations in the last year: 0.09 (68.4% of exposed subjects in cases vs. 63.3%% in control group), >4 ER visits in the last year: 0.82 (75.6% of exposed subjects in cases vs. 60%% in control group), BDR: 0.63 (69.0 of exposed subjects in cases vs. 59.3% in control group).

Characteristics of the study population are shown in Table 1. *Ascaris* sensitization was frequent (62.3%, 95% CI: 56.8 - 67.8), more common in males and inversely proportional to socio-economical stratum. Also, it was associated with atopy, especially with a positive SPT to any of the two HDM species. Prevalence of active ascariasis was low (3.8%, 95% CI: 1.7 – 6.0, mean of intensity: 19.332 e.p.g., range: 78 to 156.000). *Trichuris trichuria* infection was less frequent (1.9%, 95% CI: 0.4 – 3.4). *Ascaris* sensitization was positively associated with *Ascaris* infection (Fisher exact test: p = 0.04) or self-reported helminth expulsion in the past (χ^2^: 4.20, p = 0.04). Infected patients had higher *Ascaris*-specific IgE levels than those with a negative stool examination (p = 0.01). Furthermore, IgE sensitization to Asc s 1 was high (70.3%, 95% CI: 65.1 – 75.5).

**Table 1:** **Socio-demographic characteristics of the study population**

		***Ascaris*** **sensitization**		
**Variables**	**All patients**	**Yes (n = 195)**	**No (n = 118)**	**p-value**
**Gender (male)**	28.1 (23.0- 33.3)	36.9 (29.9 - 44.0)	13.6 (7.0 - 20.2)	<0.0001
**Age (mean ± SD)**	32.9 ± 16.9	35.96 ± 15.7	31.1 ± 17.3	0.01
Socio-economical strata*				
**1**	62.3 (56.8 - 67.8)	67.7 (60.9-74.5)	53.4. (44.0-62.8)	
**2**	31.6 (26.3 - 36.9)	29.2 (22.6-35.9)	35.6. (26.5-44.7)	<0.01
**3**	6.1(3.3 - 8.9)	3.1 (0.4-5.8)	11.0 (4.9-17.1)	
**Smoking habit**	5.1 (2.5-7.7)	3.4 (0.6-5.2)	6.2 (4.3 - 16.1)	0.42
**Co-habitation with a smoker**	26.5 (21.5-31-6)	30.8 (24.0-37.5)	19.5 (11.9 – 27.1)	0.02
**Current** ***Ascaris*** **infection**	3.8 (1.7 – 6.0)	6.2 (2.5-9.8)	0 (0)	0.04
**Antecedent of worm expulsion**	62.3 (56.8 – 67.8)	66.7 (59.8 – 73.5)	55.1 (45.7 – 64.5)	0.04
**Allergic rhinitis**	85.6 (81.6 - 89.7)	87.2 (82.2 - 92.1)	83.1 (75.9 - 90.2)	0.31
**Atopy (>1 positive SPT)**	79.9 (74.4 – 85.4)	84.2 (77.8 – 90.6)	72.9 (62.9 – 83.0)	0.04
**Sensitization to** ***B. tropicalis***	67.4 (62.1 72.8)	84.6 (79.3 - 90.0)	39.0 (29.8 - 48.2)	<0.0001
**Sensitization to** ***D. pteronyssinus***	60.7 (55.1 - 66.3)	75.4 (69.1 – 81.7)	36.4 (27.3-45.6)	<0.0001
**Total IgE ‡†**	349.436 ± 1177.4	585.4 ± 1412.1	149.0 ± 201.3	<0.0001
**Specific IgE to** ***B. tropicalis*** **†**	2.37 ± 30.9	11.98 ± 34.99	0.13 ± 15.52	<0.0001
**Specific IgE to** ***D. pteronyssinus*** **†**	0.94 ± 34.6	4.04 ± 39.27	0.09 ± 21.12	<0.0001

Frequency rates (%) for categorical variables are shown. Their 95% confident intervals are shown in parenthesis.

*Trend analysis.

‡ Geometric mean (standard deviation of mean) are reported.

†Comparison by Mann–Whitney U Test.

### Most patients reported symptoms of uncontrolled asthma

Atopic asthma was common (76.5%, 95% CI: 71.6 – 81.4) and indicators of uncontrolled asthma were frequently observed (Table 2). Most patients used short-acting β2 receptor agonist as a “control medication” (79.9%, 95% CI: 75.3 – 84.5). Current use of oral corticosteroids was more common (42.6%, 95% CI: 36.9 – 48.3) than that of inhaled corticosteroids (31.5%, 95% CI: 26.1 – 36.8). There were no significant differences in the use of these drugs among socioeconomic strata, except for oral corticosteroids, whose usage was more frequent in the poorest stratum (trend test: 5.72, p = 0.02); those belonging to stratum 1 used oral corticosteroids 2.19 more times (95% CI: 1.33 – 3.63) than the other strata (stratum 1 vs. 2 + 3).

**Table 2:** **Clinical indicators of disease severity: univariate analysis**

**Outcomes**	**All patients**	**Sensitization to** ***Ascaris***			**HDM sensitization**		
		**Yes**	**No**	**p-value**	**Yes**	**No**	**p-value**
**Episodes of severe dyspnea**	201 (64.2)	**136 (69.7)**	**65 (55.1)**	**0.01**	147 (65.3)	54 (61.4)	0.51
**Nocturnal awakenings**	272 (86.9)	169 (86.7)	103 (87.3)	0.53	**201 (89.3)**	**71 (80.7)**	**0.04**
**> 4 ER visits in the last year**	78 (24.9)	**58 (29.7)**	**20 (16.9)**	**0.01**	59 (26.2)	19 (21.8)	0.34
**Hospitalizations in the last year**	38 (12.1)	25 (12.8)	13 (11.1)	0.53	30 (13.3)	8 (9.1)	0.30

Significant associations are in bold.

### Ascaris and house dust mite sensitization were associated to indicators of asthma severity

We analyzed the relationship of *Ascaris* and HDM sensitization with clinical indicators of asthma severity (Table 2). After adjusting by covariates, sensitization to *Ascaris* was associated with “having episodes of severe dyspnea” and “ER attendance >4 times during the last year”, even when HDM sensitization was included in the model (Table 3, Model 2). After stratification by atopy, the relationship of *Ascaris* sensitization with “ER attendance >4 times during the last year” only remained significant in atopic subjects (aOR: 4.07, 95% CI: 1.14 – 14.49; p = 0.03). HDM sensitization was associated with “having nocturnal awakenings” (aOR: 2.09 95% CI: 1.03 - 4.28, p = 0.04), independently of *Ascaris* sensitization (aOR: 2.68, 95% CI: 1.21 - 5.95, p = 0.02).

**Table 3:** ***Ascaris*** **sensitization and indicators of asthma severity: multivariate logistic regression analysis**

**Outcomes**	**Model 1**			**Model 2**		
	**OR**	**95% CI**	**p-value**	**OR**	**95% CI**	**p-value**
**Episodes of severe dyspnea**	1.90	1.13 - 3.18	0.02	1.90	1.08 - 3.34	0.03
**> 4 ER visits in the last year**	2.31	1.26 - 4.22	0.01	2.23	1.15 - 4.30	0.02
**Bronchodilator responsiveness**	1.87	1.05 - 3.31	0.03	1.36	0.73 - 2.52	0.33

Model 1: Adjusted by age, gender, socioeconomic strata and tobacco exposition.

Model 2: Model 1 plus HDM sensitization.

Regarding medication for asthma control, use of short-action β_2_ agonist inhalers was associated with *Ascaris* sensitization (aOR: 1.86, 95% CI: 1.01-3.48, p = 0.05), the significance was stronger in the sub-group of HDM-sensitized patients (aOR: 2.59 95% CI: 1.19 - 5.62, p = 0.016). There were no differences in the use of inhaled or oral corticosteroids according to *Ascaris* sensitization. HDM sensitization was neither associated to any of these outcomes.

We also evaluated the effect of sensitization to molecular components from *Ascaris* (Asc s 1 and Asc l 3) and HDM (Blo t 5, Blo t 10, Der p 2 and Der p 10) on indicators of asthma severity. Sensitization to Blo t 5 (aOR: 1.93 95% CI: 1.12-3.33, p = 0.02), Asc s 1 (aOR: 2.47 95% CI: 1.10 – 5.71, p = 0.03), Der p 10 (aOR: 2.44 95% CI: 1.19 – 4.98, p = 0.01) and Asc l 3 (aOR: 2.23 95% CI: 1.10 – 4.50, p = 0.02) were significantly associated with ER attendance > 4 times in the last year after adjustment by age, gender, tobacco exposure, socio-economical strata and atopy. No significant associations were found among sensitization to Der p 2 and any indicator of asthma severity (data not shown).

### HDM sensitization was associated with bronchodilator response

Descriptive information about lung function is shown in Table 4. Neither *Ascaris*, nor HDM sensitization were associated to predicted basal or post bronchodilator (BD) FEV1 values or FEV1/FVC indexes; however, BDR was significantly associated with HDM sensitization. In multivariate analysis, after adjustment by gender, age and cigarette exposition, *Ascaris* sensitization was also associated to this outcome (aOR: 1.87, 95% CI: 1.05 – 3.31; p = 0.03) however, the significance disappeared after including HDM-sensitization in the model. In contrast, HDM sensitization was associated with BDR independently of *Ascaris* sensitization (aOR: 2.67, 95% CI: 1.33 - 5.32, p = 0.01). Furthermore, a significant association was also observed with Der p 2 sensitization (aOR: 2.24: 1.25-4.02, p = 0.01) but not with other recombinant allergens. Since BDR has been associated with indicators of severity [43] we further explored this relationship in our population, finding that it was associated with ER attendance (χ^2^ = 6.44, p = 0.01) and nocturnal symptoms (χ^2^ = 8.80, p = 0.003).

**Table 4:** **Influence of HDM or** ***Ascaris*** **sensitization on lung function parameters**

**Outcomes**	**All patients (n = 262)**	**Sensitization to** ***Ascaris***			**HDM sensitization**		
		**Yes**	**No**	**p-value**	**Yes**	**No**	**p-value**
**Continuous variables (mean ± SD)**							
**Predicted baseline FEV**_**1**_ **(%)**	71.40 ± 20.61	70.38 ± 20.50	73.02 ± 20.75	0.31	71.03 ± 20.78	72.38 ± 20.27	0.64
**Predicted post BD FEV**_**1**_ **(%)**	77.70 ± 18.60	77.30 ± 18.11	78.32 ± 19.41	0.67	77.89 ± 18.60	77.21 ± 18.70	0.79
**FEV1/FVC (%)**	86.08 ± 15.80	85.42 ± 16.07	87.14 ± 15.37	0.39	85.17 ± 15.88	88.48 ± 15.42	0.13
**Categorical [n (%)]**							
**Predicted baseline FEV1 < 80%**	158 (60.31)	100 (62.5)	58 (56.9)	0.36	117 (61.6)	41 (56.9)	0.49
**Bronchodilator responsiveness**	100 (38.2)	67 (34.4)	33 (28.0)	0.11	81 (42.6)	19 (26.4)	**0.01**

### IgE response to Ascaris correlates with house dust mite specific IgE

**Figure 2:**
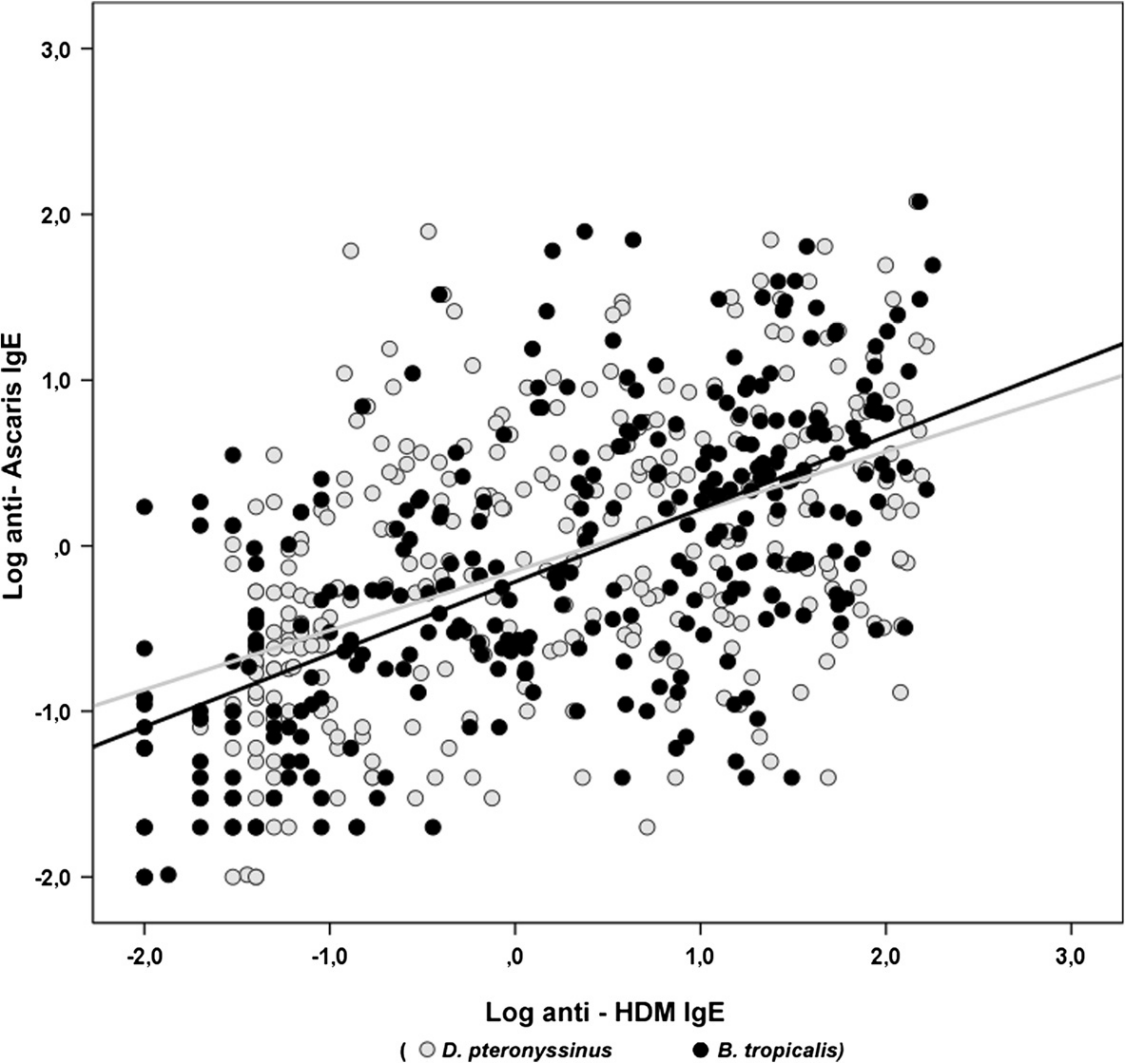
**Correlation among** ***Ascaris*** **,** ***B. tropicalis*** **and** ***D. pteronyssinus*** **- specific IgE values.** Log transformed values were represented in this superimposed dispersion plot; linear regression slopes for *B. tropicalis – Ascaris* (black) and *D. pteronyssinus* – *Ascaris* (grey) [X-Y] pairs are also shown.

*Ascaris* specific IgE values were highly correlated with those against *B. tropicalis* (Spearman rho: 0.63, p < 0.001) or *D. pteronyssinus* (Spearman rho: 0.53 p < 0.001) (Figure 2). The IgE response to the highly cross-reactive tropomyosin allergen group was also explored. Sensitization rates to the tropomyosins from *Ascaris* (Asc l 3: 24.0%, 95% CI: 19.1 – 28.9) and HDM were similar (Blo t 10: 26.4% 95% CI: 21.3 - 31.5, Der p 10: 26.1% 21.0-31.1). Specific IgE values among the three allergens were highly correlated (Spearman rho coefficients: Asc l 3 – Der p 10 = 0.58, Asc l 3 – Blo t 10 = 0.57, Blo t 10 – Der p 10 = 0.85, p < 0.001 in all cases) but they were significantly higher for HDM tropomyosins.

### *Ascaris* sensitization influences IgE responses to mite specific allergens

**Figure 3:**
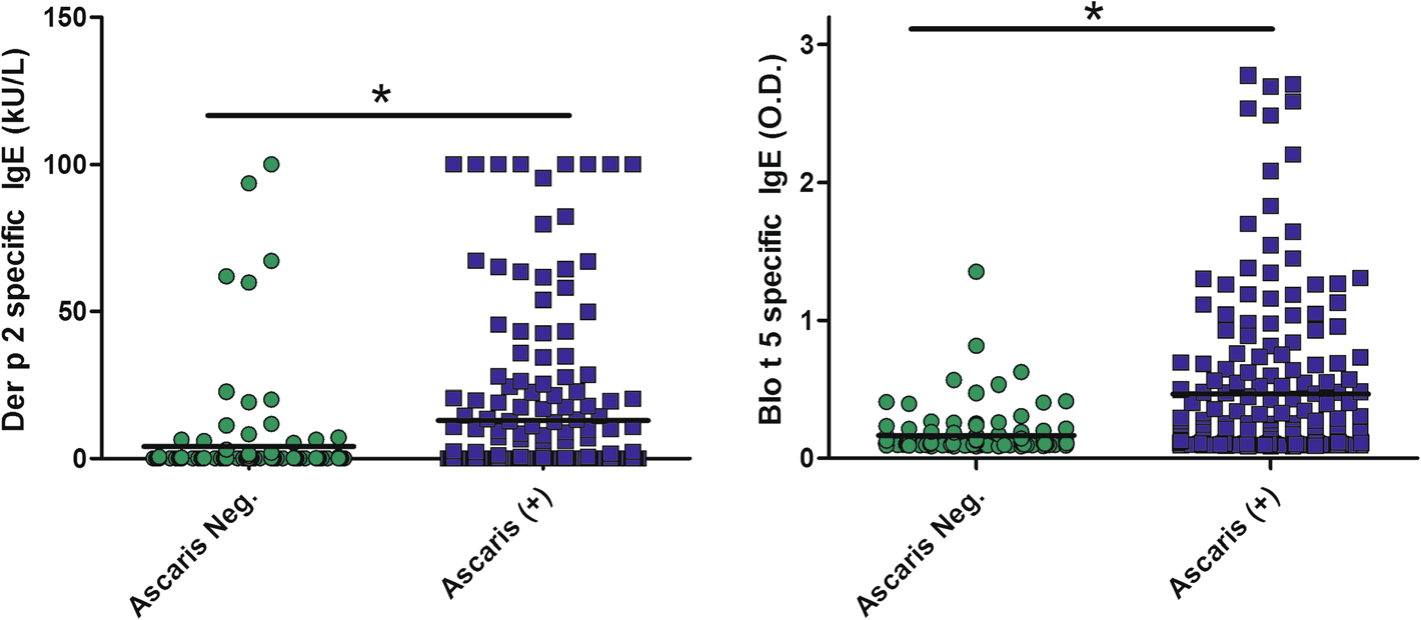
**IgE responses to HDM-specific allergens according to Ascaris sensitization.** IgE response to Der p 2, a specific marker for *Dermatophagoides spp.* and Blo t 5, a specific marker for *Blomia tropicalis* among Ascaris sensitized and non sensitized (Ascaris neg.) subjects. * p < 0.001.

As shown in Figure 3, significantly higher IgE levels to HDM specific markers were found in *Ascaris* sensitized patients compared to non-sensitized subjects (Blo t 5: 0.47 ± 0.58 vs. 0.17 ± 0.16 OD, p < 0.001; Der p 2: 12.90 ± 25.52 vs. 3.54 ± 13.80 kU/L, p < 0.001).

## Discussion

In this work, we investigated the effects of the IgE response to *Ascaris* on the severity of asthma symptoms in patients from a socio-economically deprived population of the tropics. Asthma was mainly atopic and indicators of uncontrolled disease, as well as the inappropriate use of control medication, were very frequent. In spite of a low prevalence of current *Ascaris* infection (as detected by stool examination), IgE sensitization to this helminth was high and associated to poverty. Also, it was more frequent in males, a finding previously described [12]. Because of co-exposure and their cross-reactivity [25,36] anti-*Ascaris* specific IgE levels and those against HDM were highly correlated. As in other reports, *Ascaris* sensitization was associated with different clinical indicators of asthma severity [10,12,20], but importantly, this is the first study evaluating the antibody responses with purified allergens, further supporting the results obtained with the complete extracts.

Anti-*Ascaris* IgE response was used as an indicator of infection because it is well known that diagnosis of ascariasis by stool examination, especially in places with low-burden infections, is limited by its low sensitivity [44,45]. Anti-*Ascaris* and anti-Asc s 1 IgE antibodies may identify cases of current infection as well as previous exposure [46] and our main interest was to evaluate the relationship of this IgE response with indicators of asthma severity. The low rate of positive stool examination precluded an analysis of its association with disease severity.

Clinical indicators of asthma severity were very frequent in this population and this could be related to low level of asthma control, as suggested by the underuse of appropriate treatment [47]. *Ascaris* sensitization was associated with increased odds of suffering severe dyspnea episodes and ER attendance, but it was also significantly related to poverty. Therefore, it is possible that socio-economic conditions might explain the observed results rather than a direct biological effect of ascariasis on symptoms severity, however, this variable was not significantly associated with any of the indicators of disease severity*.* Moreover, there were no differences in the use of control medication among socio-economical strata, but use of oral corticosteroids was more common among the poorest. Accordingly, the relationship of *Ascaris* sensitization with the severity of symptoms was independent of medication access due to socio-economic conditions.

Cross reactive IgE response to mites and helminths, as well as co-exposure to both sources may be confounding factors when assessing the relationship between ascariasis and allergic diseases [25,36]. For example, there is usually strong co-linearity between sensitization to *Ascaris* and HDM. It has been argued that adjusting for HDM sensitization in the multivariate logistic regression model when analyzing the effects of *Ascaris* sensitization is too conservative [12]. However, in our study, most associations between *Ascaris* sensitization and asthma severity remained significant after adjusting by HDM sensitization. Also, when using the nematode specific molecular component Asc s 1, multivariate analysis showed a significant association with ER attendance.

Previous studies have found that *Ascaris* sensitization is a risk factor for increased asthma severity or related outcomes [12,20]. Deworming of *Ascaris*-infected children was associated with improvement of asthma symptoms and reduction of controller therapy [48]. In children from rural areas of Venezuela the percentage of FEV1 predictive values (a marker of disease severity) correlated inversely with anti-*A. lumbricoides* IgE levels [10]. Hunninghake*et al.* also found significant associations between *Ascaris* sensitization and indicators of asthma severity (airway responsiveness, hospitalizations and BDR) in children from Costa Rica living in an urban setting with low burden of ascariasis; however, only the association with BDR was significant after adjustment by HDM sensitization [12]. In contrast, in our study, associations were found with ER attendance, severe dyspnea episodes and BDR, the latter, non significant after correction.

There are reasons to think that *Ascaris* sensitization may increase severity of symptoms in asthmatic patients. Several experimental and epidemiological findings [14,15] support that ascariasis may enhance the IgE response, not only to parasite-derived antigens but also to bystander antigens [49], such as aeroallergens [50]. In fact, our study shows that higher IgE levels to the HDM-specific allergens Der p 2 and Blo t 5 were found in *Ascaris* sensitized patients. Therefore, it is possible that exposure to *Ascaris* promotes allergic responses and in turn asthmatic symptoms by increasing Th2 cytokine production [51] as well as IgE synthesis to parasite components and environmental allergens [52,53]. The induction of parasite-specific IgE antibodies may have biological impact through specific or cross-reactive antibody responses. Supporting this idea, sensitization to Asc s 1, but also to *Ascaris* and HDM tropomyosins were identified as risk factors for ER attendance. It is expected that these promoting effects operate especially in individuals genetically predisposed to develop IgE-mediated inflammation, which could explain why the relationship of *Ascaris* sensitization with ER attendance only maintained it significance in the group of atopics.

During active infection, migration of *Ascaris* to the lungs may also increase the severity of asthma symptoms. Direct exposure to parasite allergens expressed during the larval stage that reach the lungs (e.g. Asc l 3 and Asc s 1) [31,54] may trigger asthma exacerbations through IgE-dependent mechanisms. The direct effect on pro-inflammatory cytokines on the airways should also be considered because epithelial damage induced by parasite invasion causes overexpression of some “alarmin” cytokines that may promote the Th2 responses [55,56] and asthma severity [57]. For example, it has been shown that stimulation of peripheral blood monocyte cells with *Ascaris* antigens induces IL-17 and IL-33 production in parasite-exposed individuals [58]. There is no certainty about when immune response totally eliminate *Ascaris* infection in humans, but experimental data from immunized pigs exposed to *Ascaris* eggs indicate that the infection is controlled at an early stage (L2-L3 larvae) by an effective immune response that kill the parasite in the intestinal mucosa. This inflammatory response was characterized by cellular infiltration of eosinophils and mast cells and accompanied by over expression of Th2 cytokines in the intestine [59]. This could happen in humans, inducing systemic responses even when the larvae do not reach the lung.

In regard to lung function the IgE response to *Ascaris* was only associated to BDR, however it did not remain significant after correction by HDM sensitization. Other studies have shown that BDR correlates with eosinophilic inflammation, atopy and bronchial hyper reactivity [43,60] and is associated to a good response to controller therapy [61]. We found that BDR was related to some indicators of asthma severity but it seems that HDM allergy is the main determinant of this phenotype.

Several limitations of this study should be discussed. Although asthma diagnosis was done with standard clinical and functional pulmonary tests, in some cases spirometry could not be done, then, power of the study was not sufficient to detect possible associations of *Ascaris* IgE with lung function parameters. In addition, we only explored the association between *Ascaris* sensitization and clinical indicators of asthma severity, the use of biological markers would have been of help to better characterize the severity of inflammation.

## Conclusions

In this population of the tropics, IgE sensitization to *Ascaris* and cross-reactive tropomyosins was frequent and associated with clinical indicators of asthma severity. The significant relationship between sensitization to the nematode-specific marker Asc s 1 and ER attendance supports these findings. Moreover, ascariasis seems to increase the IgE responses to mite specific allergens in humans.

## Abbreviations

HDM: House dust mites
ER: Emergency room
SPT: Skin prick test
FEV1: Forced expiratory volume in one second
FVC: Forced vital capacity
e.p.g.: Egg per gram of feces
OD: Optical density
BDR: Bronchodilator responsiveness
